# Developing, purchasing, implementing and monitoring AI tools in radiology: practical considerations. A multi-society statement from the ACR, CAR, ESR, RANZCR & RSNA

**DOI:** 10.1186/s13244-023-01541-3

**Published:** 2024-01-22

**Authors:** Adrian P. Brady, Bibb Allen, Jaron Chong, Elmar Kotter, Nina Kottler, John Mongan, Lauren Oakden-Rayner, Daniel Pinto dos Santos, An Tang, Christoph Wald, John Slavotinek

**Affiliations:** 1 University College Cork, Cork, Ireland; 2 Department of Radiology, Grandview Medical Center, Birmingham, AL USA; 3 American College of Radiology Data Science Institute, Reston, VA USA; 4 Department of Medical Imaging, Schulich School of Medicine and Dentistry, Western University, London, ON Canada; 5 Department of Diagnostic and Interventional Radiology, Medical Center, Faculty of Medicine, University of Freiburg, Freiburg, Germany; 6 Radiology Partners, El Segundo, CA USA; 7 Stanford Center for Artificial Intelligence in Medicine & Imaging, Palo Alto, CA USA; 8 Department of Radiology and Biomedical Imaging, University of California, San Francisco, USA; 9 Australian Institute for Machine Learning, University of Adelaide, Adelaide, Australia; 10 Department of Radiology, University Hospital of Cologne, Cologne, Germany; 11 Department of Radiology, University Hospital of Frankfurt, Frankfurt, Germany; 12 Department of Radiology, Radiation Oncology, and Nuclear Medicine, Université de Montréal, Montréal, Québec Canada; 13 Department of Radiology, Lahey Hospital & Medical Center, Burlington, MA USA; 14 Tufts University Medical School, Boston, MA USA; 15 Commision On Informatics, and Member, Board of Chancellors, American College of Radiology, Virginia, USA; 16 South Australia Medical Imaging, Flinders Medical Centre Adelaide, Adelaide, Australia; 17 College of Medicine and Public Health, Flinders University, Adelaide, Australia

**Keywords:** Artificial Intelligence, Radiology, Automation, Machine learning

## Abstract

Artificial Intelligence (AI) carries the potential for unprecedented disruption in radiology, with possible positive and negative consequences. The integration of AI in radiology holds the potential to revolutionize healthcare practices by advancing diagnosis, quantification, and management of multiple medical conditions. Nevertheless, the ever-growing availability of AI tools in radiology highlights an increasing need to critically evaluate claims for its utility and to differentiate safe product offerings from potentially harmful, or fundamentally unhelpful ones.

This multi-society paper, presenting the views of Radiology Societies in the USA, Canada, Europe, Australia, and New Zealand, defines the potential practical problems and ethical issues surrounding the incorporation of AI into radiological practice. In addition to delineating the main points of concern that developers, regulators, and purchasers of AI tools should consider prior to their introduction into clinical practice, this statement also suggests methods to monitor their stability and safety in clinical use, and their suitability for possible autonomous function. This statement is intended to serve as a useful summary of the practical issues which should be considered by all parties involved in the development of radiology AI resources, and their implementation as clinical tools.

**Key points** • The incorporation of artificial intelligence (AI) in radiological practice demands increased monitoring of its utility and safety.

• Cooperation between developers, clinicians, and regulators will allow all involved to address ethical issues and monitor AI performance.

• AI can fulfil its promise to advance patient well-being if all steps from development to integration in healthcare are rigorously evaluated.

## Section 1: Introduction

Artificial Intelligence (AI) is likely to be the single most-disruptive influence on radiology in many decades, and potentially since the very beginnings of our specialty. Previous new technologies disrupted practice by introducing new capabilities, with greater capacity to identify disease and differentiate tissues. These functioned as natural extensions of already-existing ways of doing things; older, less-effective techniques were supplanted, replaced by new modalities with greater effectiveness. All of these changes took place within the same milieu of human radiologists utilising the available tools for the benefit of patients. The tools changed, the work-patterns remained fundamentally similar.

Artificial intelligence offers the possibility of change that goes far beyond previous disruptions. Its champions have sometimes suggested that AI can replace radiologists entirely [1], although some have subsequently revised their views and come to see dangers in uncontrolled AI development [2]. More realistically, AI is increasingly being researched as a potential adjunct to radiologist-led interpretation of imaging [3]. Research is also being directed towards AI replacing traditional roles of radiologists, including study and protocol selection [4], and direct generation of radiology reports by AI models [5].

In the midst of the burgeoning literature, publicity and claims surrounding AI in radiology, how is a radiologist, practice manager or software purchaser to winnow the wheat from the chaff, to critically evaluate claims of utility and benefit from AI utilisation, to differentiate fully-evaluated and safe product offerings from those with potential to function other than as advertised, or, worse, to do harm? In this multi-society paper, representatives of the American College of Radiology (ACR), Canadian Association of Radiologists (CAR), European Society of Radiology (ESR), Royal Australian and New Zealand College of Radiologists (RANZCR), and Radiological Society of North America (RSNA) attempt to define the specific potential problems around AI incorporation into radiological practice, the relevant ethical issues that arise, the considerations that should be borne in mind by developers of AI tools, the issues that should be considered by those authorised to license or certify AI tools for clinical use, how AI tools should be evaluated by purchasers and users when considering their introduction into clinical practice, how they should be monitored for long-term stability and safety, and how we should evaluate their suitability for autonomous function.

## Section 2: What is the problem?

### A. Why do AI algorithms differ from previous IT/informatics developments in radiology?

Traditional computer-aided detection (CADe) or diagnosis (CADx) systems as used in radiology for about 30 years are rule-based, using classical machine learning techniques with handcrafted features. The features the system was intended to detect, such as shape, size or texture of a lesion, were manually pre-defined, and then used to detect abnormalities in radiological images [6]. Although useful, CAD was limited by the need for manual feature engineering and the inability to learn and adapt over time.

Modern AI algorithms, particularly those based on deep learning, fundamentally differ from traditional CAD by automatically learning relevant features from data without explicit definition and programming. Deep learning algorithms can learn to identify patterns in radiological images by being trained on large datasets and, in principle, can continuously learn and improve their performance as they are exposed to more data [7]. Training of deep learning models can use either supervised learning (most used today, presenting pairs of inputs and desired outputs), unsupervised learning (the system clusters the data in classes), or reinforcement learning (the system learns by being rewarded or punished) [8].

Another key difference is the level of automation that AI algorithms can bring to radiology. While traditional CAD systems can assist in the detection of abnormalities, AI algorithms have the potential to automate many routine radiology tasks, such as image segmentation and measurement, image quality and completeness evaluation, and can provide decision support by analyzing a vast amount of data in real time [9, 10].

The implementation of AI in radiology presents new challenges, such as the need for large annotated datasets for training AI algorithms, ensuring the transparency and interpretability of AI decisions, and addressing ethical and regulatory considerations [11, 12].

### B. Why do we need to evaluate AI models in new ways before they enter routine clinical use?

Most AI models in Radiology are used to support lesion detection or quantification, or to help radiologists’ decision making [13]. Some newer approaches also help with analysing patients’ history or with writing reports and/or impressions of examinations [14]. To ensure safe operation of AI models in Radiology, it is essential to educate radiologists and other potential end-users about the principles of AI and teach them the limits and potential risks when using AI models [15, 16].

It is also important to evaluate the accuracy of AI models on the target population before introducing them into clinical practice, and after that introduction, their performance should be monitored to detect drifts in accuracy.

The integration of AI algorithms into the radiology workflow is key to ensure their safe and consistent operation. The lack of widely accepted standards for AI integration is still a challenge [17]. In this context, attention should be paid to the interface design. Exposing radiologists to an increasing number of complex interfaces is undesirable, and is liable to diminish utility and acceptance of AI tools [18].

### C. How can we differentiate among the multiplicity of products on offer?

The integration of AI in radiology has the potential to revolutionize healthcare practices, offering advanced solutions to diagnose, quantify, and manage multiple medical conditions. However, the evaluation of AI models extends beyond clinical accuracy, encompassing business and technical considerations. These, and other aspects of how potential users and purchasers can evaluate AI tools before implementation, are explored in detail in Section 6.

## Section 3: What are the ethical issues?

Medical ethics is underpinned by four underlying principles:

1. Beneficence (doing good)
2. Non-maleficence (doing no harm)
3. Autonomy (patient freedom to choose)
4. Justice (ensuring fairness) [19–22].

These principles apply to medical practice in the broadest sense and therefore encompass ethical deliberations pertinent to AI in radiology. This section draws upon work by multiple stakeholders that include the AAPM, ACR, CAR, ESR, EuSoMII, RANZCR, RSNA, and SIIM [11, 23–28] and considers ethical issues that arise in the context of development, deployment, use and monitoring of AI systems.

In 2019, the majority of the above societies collaborated on a multisociety statement on Ethics of AI in Radiology [23], delivering the following key messages:

- AI in radiology should promote well-being, minimize harm, and ensure that the benefits and harms are distributed among stakeholders in a just manner.
- AI should respect human rights and freedoms, including dignity and privacy. It should be designed for maximum transparency and dependability.
- Ultimate responsibility and accountability for AI remains with its human designers and operators.
- The radiology community should develop codes of ethics and practice for AI that promote any use that helps patients and the common good, and block use of radiology data and algorithms for financial gain without those two attributes.
- There is a need for extensive research to understand how to best deploy AI in clinical practice.
- AI carries potential pitfalls and inherent biases. Widespread use of AI-based intelligent and autonomous systems in radiology can increase the risk of systemic errors with high consequence, and highlights complex ethical and societal issues.

### Key statement

AI in radiology should promote well-being, minimize harm, respect human rights such as dignity and privacy, and ensure that benefits and harms are distributed among stakeholders in a just manner.

Given the critical dependency of AI upon data, ethical issues relating to acquisition, use, storage and disposal of data are central to patient safety and the appropriate use of AI. Important ethical issues relate to consent, privacy and data protection, data ownership, bias and fairness, transparency and integration of AI into clinical practice [11, 23].

#### Privacy, consent and data ownership

AI systems in radiology require access to large amounts of patient data for training and operation. Ensuring that this data is used ethically involves maintaining patient privacy, obtaining informed consent for data use, and ensuring data security. Multiple factors impinging upon ownership of patient data include relevant legislation, patient privacy and autonomy, broader public interest, health care provider and AI developer interests, and copyright issues [23, 24]. Inevitably, different countries vary with regard to these influences and this may make use of data by developers and others even more complex. In principle, decisions regarding the extent of patient consent required (‘informed’, ‘opt-out’ or ‘presumed’) reflect the balance between potential societal benefit or beneficence and patient autonomy. The anonymity of patient data is also an important but complex consideration and, if not maintained, is another source of risk. Potential harms, such as discrimination, insurance costs and humiliation, must be considered when data-related decisions are made.

#### Bias and fairness

AI systems can unintentionally perpetuate or even amplify existing biases in healthcare, leading to unfair outcomes (Table 1). In particular, AI systems rely on training data, lack context and are more likely to exhibit bias if the data used to train the AI system are not representative of the patient population on which the AI system will be used. This bias is due to differences in populations, and may reflect gender, sexual orientation, ethnicity, social, environmental, or economic factors. The data utilised may also contain inherent biases for other reasons, such as bias derived from the humans who label data for training the AI system. Different scanning devices and protocols may also influence the data used during AI development and induce bias.

**Table 1:** Typology of biases

Type of bias	Explanation
Data bias	Bias can occur with any dataset. Common sources of bias potentially promote or harm group-level subsets based on gender, sexual orientation, ethnic, social, environmental, or economic factors
Clinical confounding bias	Radiology AI may be biased by clinically confounding attributes such as comorbidities
Technical bias	Bias can be introduced due to subtle differences in raw and post-processed data that come from different scanning techniques
Automation bias	This is the tendency for humans to favor AI decisions, ignoring contrary data or conflicting human decisions. This can lead to errors of omission (when humans fail to notice, or disregard, the failure of an AI tool) and commission (when one erroneously accepts or implements a machine’s decision despite other evidence to the contrary). (See also Section 8)

The interaction between AI systems and humans is also germane. Humans have an appreciation of context and are more likely to understand if AI outputs are inappropriate in a given clinical context and act rather than simply accepting incorrect AI advice. In contradistinction, automation bias is the tendency of humans to favor the decisions of AI systems over human decisions, which can lead to errors if the AI system is incorrect. This automation bias may be accentuated when a radiologist is fatigued or there is a limited radiology workforce and therefore limited capacity to supervise AI output. Risks to patient safety will also be higher when autonomous AI systems are implemented or the AI system continues to learn and adapt over time. In these situations, the need for assessment and monitoring of AI system performance becomes commensurately greater.

### Key statement

AI systems rely on training data, lack context and are more likely to exhibit bias if the data used to train the AI system are not representative of the patient population on which the AI system is used.

#### Transparency and explainability

Transparency requires provision of clear information about an AI system’s capabilities and limitations, in particular the purpose for which the systems are intended, the conditions under which they can be expected to function as intended and the expected level of accuracy in achieving the specified purpose. This information is important especially for deployers of the systems, but it may also be relevant to competent authorities and affected parties [29]. The concept of transparency should also extend to patients being made aware if AI systems are being used.

Many deep learning AI systems work as "black boxes", and in this setting radiologists and other healthcare providers may have little or no insight into how the AI algorithm arrived at its conclusions. Although difficult to achieve with some deep learning systems, provision of information about how decisions are made results in greater comprehensibility and trust amongst patients and medical professionals. Definitions vary, but transparency, interpretability (the ability to understand the workings of an AI system) and explainability (how an AI system makes decisions and presents its output in detail) are desirable, but come with risks. Opinions differ, but the need for transparency, interpretability and explainability should be balanced against potential harm relating to loss of privacy, loss of proprietary information and malicious attacks.

#### AI in clinical practice

Access to data, various skills and computing power is vital during development and deployment of AI systems. These resources are not evenly available, leading to potential inequity of access to benefits from AI, resulting from multiple factors that include geographic location, ethnicity and availability of financial resources. For example, resource-rich countries or hospitals may have access to more advanced AI tools than their resource-poor counterparts, thus exacerbating health disparities.

The introduction of AI into healthcare could alter the dynamic between physicians and patients, with potential implications for patient trust. Historically, clinicians are held responsible when an acceptable standard of care is not met. Where an AI system is used and the standard of care is not met, accountability and liability may extend to the developer and to the healthcare entity that implemented the AI system in addition to the clinician [11, 23].

Conflicts of interest may also arise where radiologists, other healthcare professionals or healthcare systems are engaged by or otherwise involved with commercial entities marketing AI systems [28]. In order to achieve optimal performance and patient safety, consideration must be given to successful integration of AI systems into workflow and with other technology, and education of those using such systems. Done right, AI implementation stands to benefit the patients & public, and radiologists are well advised to stay relevant by leveraging their professional skills to promote safe and effective AI deployment [11, 23].

### Key statement

Addressing ethical issues in AI will require a combination of technical solutions, government activity, regulatory oversight, and ethical guidelines developed in collaboration with a wide range of stakeholders, including clinicians, patients, AI developers, and ethicists.

## Section 4: What should developers consider when creating a new AI tool for radiology?

### A. Clinical utility of new products

New products should improve the quality or efficiency of existing workflows in terms of lesion detection, segmentation, diagnosis, or prediction of clinical outcomes. A common mistake among radiology AI developers is the development of solutions reflective of available technology and datasets, rather than those with clinical utility supporting existing workflows. Society-developed resources, such as the ACR DSI Define-AI directory, often serve as a good starting place to ensure the technology being developed meets genuine clinical needs [30]. In the absence of an existing Use Case reference, or an application that is not a direct derivative evolution of an existing application, developers should involve clinicians as early as possible in the design and development process to gain insights into the feasibility and practicality of proposals, well before substantial investments in time and developer resources have been made.

#### Key statement

New products should target unmet clinical needs rather than focus on existing technology and datasets.

### B. Superiority to existing clinical/radiology tools

Demonstrating superiority over existing clinical processes can be a challenging proposition for developers, particularly those with limited clinical experience in the domain. For solutions backed by pre-existing open-science competitions, where clear performance metrics are defined and leaderboards of competition entrants are maintained, it is generally easier to demonstrate competitive equivalence or superiority. This is particularly notable in the long series of AI Challenges at annual RSNA and MICCAI conferences [31, 32]. In situations where no such open data exist, developers should determine the baseline clinical performance, and compare the AI performance with existing or approved software, or radiologist multi-reader control data. Well-designed multi-reader diagnostic accuracy studies are a common method of reporting AI solution superiority, though they can be both difficult and expensive to perform effectively. When human readers are assisted by AI, different modes of algorithm use, such as *first-reader*, *concurrent reader*, *second-reader*, or *triage* modes, may affect how relative performance is analyzed [33].

### C. Radiomics, explainability & transparency

There are certain classes of AI applications that pose particular challenges to model interpretability. *Radiomics* refers to the extraction of a large number of features from medical images using data-characterisation algorithms to describe pixel intensities, relationships between these pixels, shapes, and textures. Many of these features are non-intuitive or do not map easily to subjective or clinical image findings [34]. There has been much comment about the black box nature of AI models, with early efforts focused on heat map and saliency visualizations; some researchers have called for a combination of visualizations and generated text to improve interpretability of diagnoses [35–37]. Lessons learnt from traditional biomedical research are extremely relevant, and in situations where model transparency and explainability are poor, a higher standard of empirical evidence of performance may be required, including external or multi-centre test data to prove model generalizability, and prospective real-world evaluation, used in clinical settings that most resemble the clinical setting in which the product is most likely to be deployed.

## Section 5: What information should regulators request from developers prior to approval of AI software for clinical use?

### Key statement

Prior to approval, regulators should request information from AI software developers pertaining to the company, clinical use, implementation, product development, demonstration, cost, and publications (Table 2).

**Table 2:**
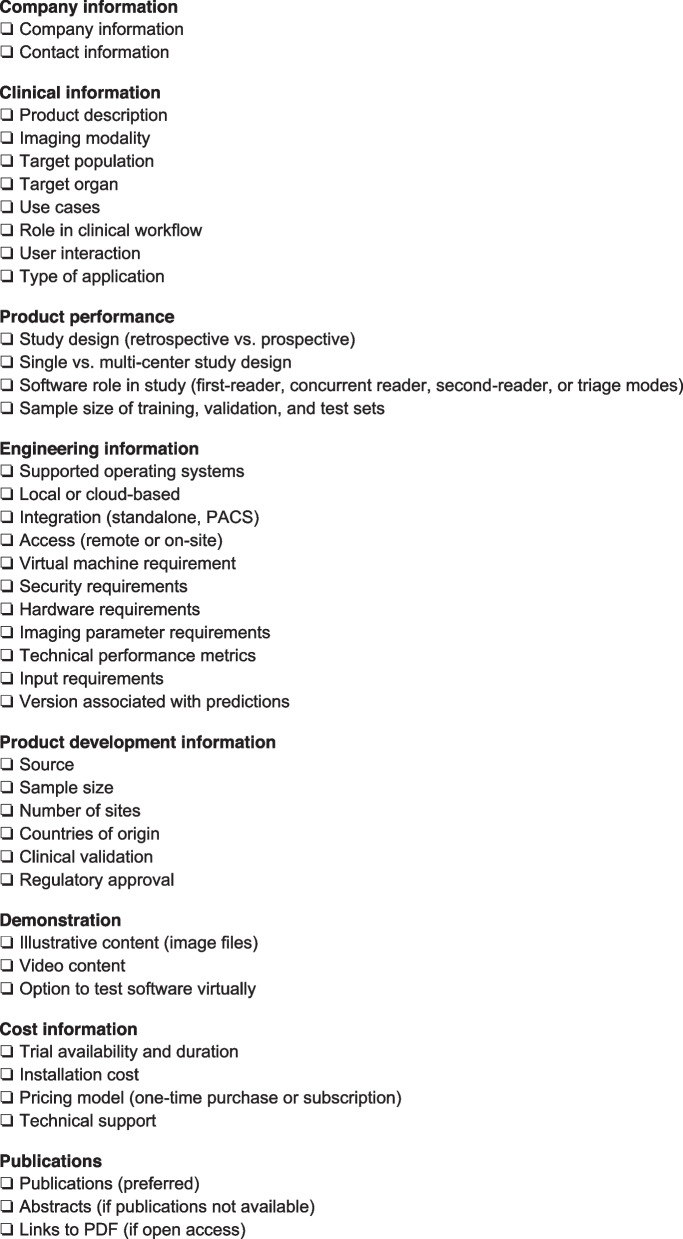
Relevant information for regulators prior to AI software assessment [adapted from [38]]

AI solutions generally present estimates of solution performance using a combination of retrospective and prospective validation trial results upon which their statements of function are based.

Regulators should pay close attention to ensure that the reported information complies with the highest standards of practice; studies should ideally adhere to criteria defined by the multiple established scientific reporting standards [39–43]. Lower quality evidence often has significant gaps in the information reported and only partially fulfills these standard criteria. Two common errors in solution performance reporting include *a failure to report a range of expected performance*—lower quality solutions often report a single summary accuracy figure—and *not reporting specific failure conditions and errors*, with lower quality solutions selectively highlighting the best diagnoses made by their systems. In the broader AI safety community, there is a strong embrace of *Model Cards* or *System Cards*, in which in-depth analyses of limitations, errors, and biases are explicitly reported, often entirely separate from the primary report of system performance [44, 45]. This level of public transparency should be strongly encouraged by regulators to foster a greater culture of AI safety, and should be a primary consideration when evaluating the quality of submissions.

Although clinical risk models differ based upon geographic jurisdiction and historical precedents, we strongly believe that any regulatory model should draw clear categories and boundaries between advisory, semi-automated, and automated systems, *with a deeper evidence base and real-world track record required for greater degrees of autonomy.* Clinical references often cite, as a relatable metaphor, automation scales that have been proposed for autonomous driving vehicles, for example the SAE J3016 Levels of Driving Automation [46]. Multiple attempts to design analogous levels of escalating automation for radiology workflow have been proposed [24, 47]. Traditional regulatory frameworks governing medical devices have focused predominantly on monitoring or therapeutic devices, which have very rarely, up until recently, exhibited any functionality with the potential of autonomous action. The general AI literature is replete with examples of negative and often unexpected harms of AI making unsupervised decisions [48]. The patient implications of decision making required in clinical medical imaging, even declarations of stability or normality, often have dramatic direct implications on patient care, which we suspect will require human co-supervision for some time.

Regulators should be particularly attuned to ensuring that solutions have an explicit *post-market quality assurance plan*. The importance of this has several aspects, but mainly relates to the issues caused by concept drift, due to changes in the patient population or occasionally even differences due to upgrades of successive new versions of AI software [49]. In practice, what this may entail is prospective performance monitoring of the AI model, for example monitoring for major deviations in month-to-month diagnostic event frequencies, with alerts raised when normal bounds are exceeded, or a control sample approach where a constant reserved held-out set of test case examples is routinely evaluated with the algorithm, to ensure no major deviations on known difficult or borderline cases [50]. At a very minimum, a clear reporting procedure for unexpected errors to the vendor, with named responsible contact personnel, should be established and made easily accessible to clinical end users.

## Section 6: What should purchasers of AI tools consider when contemplating introduction of AI tools into practice?

When contemplating the implementation of AI applications in clinical practice, various key aspects should be considered to ensure sustainable benefits to all stakeholders involved. As described in the previous section, regulatory approval from the Food and Drug Administration (FDA), the European Medicines Agency (EMA) or equivalent agencies certifies that medical devices (including AI tools) comply with the relevant regulations and have gone through a conformity assessment based on the device’s risk category. However, this certification alone does not necessarily guarantee successful implementation into clinical workflow [51]. Among other things it is therefore crucial that potential purchasers consider the following aspects:

1. What is the intended use of the AI, who will most benefit from its use, which risks are associated with its use and what is the potential economic impact?
2. How will the AI tool be integrated into the institutions’ workflows and how can the commercial claims be verified and monitored?
3. How do users need to be trained and which psychological effects need to be considered with regard to human-AI-interaction?
4. Is the FDA (or other agency) approval/clearance data reflective of accuracy on local data? Is that accuracy on local data sufficient for use in that institution and will users accept and hence engage with the AI results?

### Usage benefits, risks and cost

For any AI tool to be successfully integrated into clinical practice, stakeholders should first clearly identify areas that need improvement and define relevant key performance indicators [52, 53]. The integration of an AI tool may then be part of a larger strategy devised to attain the goal set for the institution. Alternatively, it might also be the case that a particular AI tool proposed by a vendor offers a potential to improve the quality of the institutions’ services in an area not previously considered. In either case, as outlined in Section 4A, it is essential to determine whether or not the tool solves a real, specific problem that the institution has; tools are solutions, and a solution to a non-existent problem has no value. Note also that different institutions have different problems; a tool that is valuable for one group may not have value for another.

For the positive impact of an AI tool to be measurable, objective and quantifiable goals should be set. It may be useful to consider both what proportion of cases or patients an AI tool is expected to impact, and what the magnitude of impact on each case or patient is expected to be. Purchasers should be aware that the beneficiary of the AI tools’ potential for improvement does not always need to be the radiologist or the radiology department alone. Ideally, all stakeholders involved, from the patient requiring a service to the respective institution and even the wider society could benefit from AI being successfully implemented in a clinical workflow. An example of a strong use-case could be AI as a supporting tool in high-volume radiological screening settings (e.g. mammography). In this case the benefits for patients could include earlier and better detection of breast cancer, leading to better overall outcomes, while benefits for radiologists could include increased productivity, the availability of an additional “safety net” or the potential to increase the time available for interaction with the patient [54]. Apart from improvements in productivity and service quality positively reflecting on the institution, they could potentially help reduce costs, while for the wider society positive effects on overall healthcare costs and population health could be envisioned. Such effects could also be expected for other commonly suggested use-cases, such as the detection of large vessel occlusions or in other time-sensitive situations. However, for other applications like organizational AI support tools or as-of-yet more research-driven applications (such as AI-powered opportunistic screening) the benefits might not be as easily definable [55, 56]. Depending on the local circumstances and healthcare system in place, such potential benefits need to be carefully weighed against their immediate and mid- or long-term economic impact. Return on investment (RoI) and cost–benefit analyses should be planned and carried out to ensure the viability of the planned AI integration. Depending on the healthcare system, establishment of a viable payment mechanism for AI use may be critical. AI models that primarily benefit a fee-for-service hospital or outpatient imaging center prove RoI through decreasing length of stay [57], improving throughput in the emergency department [58], increasing the volume of findings that require follow-up and/or treatment, decreasing the length of time it takes to perform an imaging exam, and improving operations in the radiology department. Other potential benefits to the radiology practice include decreased mental fatigue, improved radiologist recruitment and retention, and decreased medical malpractice liability, although these tend to be additive as they do not generally cover the cost of the AI.

Lastly, potential costs (both capital and recurrent) and risks associated with the implementation and usage of an AI system are essential components of any purchase analysis and decision. In part, risk assessment can be facilitated by consulting the risk matrix and the risk–benefit analysis provided in the regulatory files by vendors. However, some risks may not be addressed in such regulatory filings or only become apparent during use. The most obvious component of cost is the licensing costs paid to the vendor, but these are typically only a small part of the total cost of ownership. Other sources of cost include contracting and legal agreements, IT effort and professional services for integration with existing systems, training for users and administrators, infrastructure for running the AI, and ongoing maintenance and monitoring.

Other essential factors in making an informed decision include evaluating the vendor's compatibility as a reliable partner, the vendor’s staying power in a competitive environment with limited payor reimbursement (even more important in this era of AI vendor consolidation), optimized model pricing, and opportunities for collaboration beyond product purchase, such as co-development and product resale.

A key component of risk is understanding what the performance characteristics of the algorithm are likely to be in the environment in which it will be used. The error rate in use may differ substantially from what was reported in testing, particularly when the characteristics or distributions of the input data (e.g. scanner manufacturers, scan protocols, patient demographics, disease prevalence, comorbidities) differ from the test data. Ideally, each site considering implementation would perform a statistically rigorous evaluation of performance on their own local data (a method for this evaluation is presented in the Clinical Evaluation Section below). In practice, this may not be feasible. At a minimum, the characteristics of local data should be compared with those of the test data (a typical example might be where a model has been tested only on one manufacturer’s MRI scanner, but will be used on a scanner made by a different manufacturer). Where these are similar, the reported performance metrics may be relied upon with some confidence; where they are not (e.g., an algorithm tested only on adults being considered for off-label use in a pediatric hospital) one should proceed with great caution, if at all. Error frequency, conceptually the inverse of performance, is not the final word on risk, because different errors pose different risks. One should consider the detectability of the errors that are anticipated. That is, for each error, what is the probability that people in the workflow will notice that the AI has produced an erroneous output? For each detected error, what is the probability that the error will be corrected? Finally, if an error is not detected or not corrected, what is the expected impact on patients or other stakeholders? The consideration together of error frequency, detectability, correctability and impact provides a framework for assessing the direct risk of algorithmic errors. Ongoing monitoring of these risks is considered in Section 7.

Another key component of risk is the impact of an AI tool on radiologist performance. Relying on an automated tool to perform a task may lead to de-skilling of radiologists for the task the tool has taken on. This risk is particularly problematic if the radiologist is expected to perform the task manually when the tool fails, but may no longer be skilled enough to do so adequately. User over-reliance and under-reliance also decrease the accuracy of the combined output of the radiologist in combination with the AI model and is discussed further in Section 8.

A final aspect of risk that must be considered is the potential for AI to create or exacerbate healthcare disparities. AI is particularly prone to this because it is generally trained on retrospective data drawn from clinical archives, and these data represent the current and historical healthcare disparities and inequities of our society. Training an AI is a mathematical process of minimizing a cost function that proceeds without ethics or morals. Therefore AI may learn from the inequities and disparities embedded in the training data, and can perpetuate these in implementation. There is no easy or straightforward process for comprehensively identifying these biases, but it is incumbent upon us as physicians and data scientists to think about, search for and mitigate these biases; if these questions are unasked, they will most certainly remain unanswered.

### Integration, verification and monitoring

Once expected benefits and goals have been decided upon, cost–benefit analysis has been carried out and potential risks have been assessed, integration of the selected AI tools can be planned. Depending on the local IT infrastructure and policies, purchasers can consider different technical integrations—either as local installations with dedicated computational resources on site or as a cloud-based software as a service (SaaS) model. In both types of installation, data orchestration of DICOM and HL7 play a vital role ensuring the right slices from the correct series of the relevant study for the right patient in the right setting are sent to the appropriate AI in an optimized time. To achieve a robust orchestration, understanding and structuring the content of your data is essential. Unfortunately, relying on DICOM metadata is often insufficient due to the high variability and labile nature of study and series names, and the fact that DICOM headers may be incomplete. A more robust option is to use imaging AI to determine the data contents at the studies and series level and use that output for orchestration. Using computer vision AI to determine which body parts are on each image and if intravenous contrast has been administrated are two of the most useful additions. Downstream data orchestration from the AI system requires an intelligent system able to facilitate different workflows depending on an understanding of the AI results. Most current implementations only send the AI results to the Picture Archiving and Communication System (PACS). This limited integration not only allows visualization of AI results by referring physicians, which may not be optimal if these physicians haven’t been educated about the details and accuracy of the AI model, but also has been shown to increase automation bias among radiologists [59]. Furthermore, PACS currently offers limited modes for AI results integration and in most instances, the radiologist cannot modify the AI results in PACS. To optimize AI results management and integration, a PACS should enable the radiologist to interact with and modify the AI results and, if results are changed, empower the AI to immediately reprocess a new output. In addition, the updated AI result should be provided to the AI vendor so it can be used for future model improvement. This type of interaction is facilitated in a cloud-native environment where both the PACS and AI models can share radiology data and AI results. Additionally, the ability to accept and store AI results along with radiologist feedback, optimize data security, and continuously monitor AI accuracy are crucial technical aspects that are facilitated in cloud-native systems.

Whatever the integration, ideally AI tools should be well integrated into the usual clinical workflow and information systems in order to avoid additional workload by requiring users to switch between applications. A recently published survey revealed concerns about additional workload to be one of the main reasons for respondents not intending to acquire AI tools for their clinical practice [60]. The same survey found that another major concern was that the AI system would not perform as well as advertised. This concern is important and should not be overlooked. Of course, vendors will have performed testing and quality assurance of the respective AI tools during regulatory approval, but purchasers should consider validation of the AI’s performance on a local dataset, and adjust parameters if needed prior to implementation in clinical practice. This process should be repeated whenever relevant changes are made to the AI software or the equipment used in combination with the AI. In the example of a commercially available breast screening AI model an update of the AI tool resulted in a substantially different recall rate, requiring recalibration of the decision threshold to ensure continued usage with clinically acceptable diagnostic accuracy [61]. These findings highlight that it cannot be taken for granted that diagnostic performance claimed in premarket publications translates to a comparable and stable performance during clinical usage, emphasising the need for continuous post-market surveillance of the AI systems used. The exact approaches to how this should be done are currently being discussed by the respective regulatory bodies. For example, the UK’s Medicine and Healthcare products Regulatory Agency (MHRA) *Guidance for manufacturers on reporting adverse incidents involving Software as a Medical Device under the vigilance system* details various circumstances in which an adverse event should be reported—including “[failure] to identify clinically relevant brain image findings related to acute stroke” and “[degradation of MRI image] appearance of anatomical and pathological structures” [62]. Similarly, the FDA’s *Proposed Regulatory Framework for Modifications to Artificial Intelligence/Machine Learning (AI/ML)-Based Software as a Medical Device* would expect manufacturers “to commit to the principles of transparency and real-world performance monitoring” when making updates to their products [63]. Stakeholders in implementation of AI tools in clinical practice should therefore familiarize themselves with the relevant methods and metrics for clinical evaluation and devise strategies to verify performance claims prior to tool introduction, and should continuously monitor performance during routine usage [64]. This is especially important as the previously mentioned survey found that a large majority of respondents did not assess the AI’s diagnostic accuracy on a regular basis [60]. Post-market monitoring is discussed in greater detail in Section 7 (below).

### Human-AI interaction

Besides technical performance details and the practical workflow integration of AI tools in radiology, the importance of difficult-to-measure human factors should not be underestimated. AI has undeniably made impressive progress and for many use-cases can reach diagnostic performance comparable to that of human readers. This has especially been shown in the context of breast cancer screening [65–69]. However, as discussed above, many factors can influence the technical diagnostic performance of AI tools in clinical practice. While it has been suggested that the combination of human reader and AI tool could help increase overall diagnostic accuracy by either the human detecting an error made by the AI or vice versa, recent studies question this premise and highlight the need to further study the psychological phenomena that can bias decision making in a setting of human-AI interaction. It is well known that automation bias—the tendency to over-rely on automated systems, such as AI-powered decision support tools—can influence human readers and negatively impact their ability to exercise oversight [70]. Recently, a study focused on mammography found that even the most experienced readers exhibited this bias in an experimental setting and had significantly worse performance when a purported AI system suggested a wrong BI-RADS category [71]. Conversely, the opposite effect described as algorithmic aversion—where information is rejected in a decision making process solely based on it being AI-generated—can also be observed [72]. A recent study showed that radiologists and other physicians rated the same information about a chest X-ray as being less reliable when it appeared to come from an AI system than when it appeared to come from a human expert [73]. These issues are further complicated by the fact that human-AI interaction may be influenced by details of the user interface’s (UI) design. For example, while many radiologists preferred image overlays to detect pulmonary nodules, it was found that this configuration of the UI did not improve reader performance, while a minimalistic setup with text-only UI output did [74]. Similarly, a study evaluating eye gaze in endoscopy found that the usage of a computer-aided system for polyp detection led to significantly reduced eye movements while evaluating endoscopic videos and an increase of misinterpretation of normal mucosa [75]. These findings highlight the need for further education on those topics to increase awareness amongst users and stakeholders and allow for safe and successful implementation of AI into clinical routine [76]. Opportunities to help mitigate human-AI bias are discussed in Section 8. More focused research into this area is needed to provide reliable evidence on how to best design human-AI interaction.

### Clinical evaluation

While FDA or other relevant authority approval/clearance data provides some insights, testing the AI model on local data, with the local systems and workflows used in practice, is essential to ensure accuracy and relevance when the model is deployed. While local evaluation will need to be tailored to the specific AI model and local resources, Table 3 outlines tactics which may help practices decide if a given model is relevant to local practice and performs with suitable accuracy on local data (Table 3).

**Table 3:** 5-steps for assessing clinical accuracy of AI model

Step	Process	Detail
1	Review Model Accuracy	Evaluate AI model performance on local data. Look carefully at user-facing metrics (i.e., PPV and NPV) as these affect user engagement. Use this information and case-based examples to craft educational content for the radiologists to help mitigate human-AI bias
2	Calculate Optimized Enhanced Detection Rate (EDR)	EDR = (# of AI positive exams, not included in the rad report) / (# of rad reports with the identified pathology). This value represents an improvement in sensitivity and patient care that could be reached by optimally combining the radiologist and AI results
3	Identify “WOW” Cases	“WOW” cases are those that could affect patient care or hospital operations as seen through the lens of any of the radiology stakeholders including the radiologist, referring clinician, hospital administrator, patient, or payor
4	Categorize Model Pitfalls	AI models will have false positives (FP) and false negatives (FN). Try to categorize the FP and, if possible, the FN cases so these can be used to set radiologist expectations and help mitigate the human-AI bias
5	Summarize & Decide	Based on the above data, determine if the model is clinically worthwhile to roll out in your environment

A clinical accuracy evaluation process can be performed efficiently and does not require model implementation into your clinical workflow. The first step involves comparing the AI model’s performance on local data against regulatory authority documentation, specifically evaluating accuracy through the lens of radiologist acceptance and engagement with the AI tool. Hence, parameters that are radiologist-facing, including positive and negative predictive values for the disease prevalence are more relevant than overall accuracy, Area Under the Curve (AUC), or sensitivity/specificity. Secondly, calculate an “Enhanced Detection Rate,” the optimized detection that could be obtained through a combined detection of radiologist plus AI true positive results. Thirdly, impressive, or "WOW cases," should be identified to demonstrate the AI model's value to users and stakeholders. Fourthly, categorizing AI false positives and, when possible, false negative cases can set radiologist expectations and improve their acceptance of an imperfect AI model (all AI models are imperfect). Finally, all the findings should be reviewed to determine if the AI model is worthy of clinical deployment.

Ultimately, the decision lies in the balance between positive predictive value (which is highly dependent on disease prevalence) and the value and number of “WOW” cases. Radiologists are more willing to accept false positives, if the model also identifies pathology that impresses the radiologist or would add value for the patient or other stakeholder. Disease prevalence also has a strong impact on downstream model acceptance. Low disease prevalence AI models produce results with numerous false positives limiting user acceptance. Disease prevalence in a patient group presented to an AI model can be modified by properly selecting patient imaging locations, such as Emergency Department, inpatient, or outpatient. Hence, some AI models may be deployed on a subset of exams because disease prevalence in that exam subset is increased from baseline. For example, pneumothorax (PTX) on Chest XRay (CXR) has a higher prevalence in the inpatient rather than the average population. Limiting a PTX AI model to only inpatient CXRs will provide fewer false positive results and will more likely be accepted by the radiologists from an accuracy standpoint.

Utilizing information from the above 5-step clinical evaluation for radiologist education, coupled with change management, is vital to set user expectations before AI model implementation. A local AI champion plays a significant role in promoting AI adoption among radiologists. Finally, continuous user education throughout the lifecycle of AI utilization and monitoring radiologist AI usage and the combined accuracy of radiologist plus AI are instrumental in ensuring optimal patient care.

Purchasing considerations are summarised in Table 4:

**Table 4:** Purchasing considerations for AI models in radiology

Strategic	Which problem is the AI helping to solve?
	What benefit can be expected from the AI’s usage?
	How much improvement can be expected?
	Are there any risks associated with the AI’s usage? How can those be mitigated?
Regulatory	What is the AI’s intended use?
	At which risk category was the AI certified?
Performance	How can the AI’s performance be monitored?
	Can AI failures be detected and reported?
	Is performance on local data comparable to claimed performance?
	Are differences between local data and training data known?
	Does performance vary depending on the imaging device used?
	Does performance vary depending on patient characteristics (gender, ethnicity, etc.)?
Workflow	How is the AI integrated into the radiologist’s workflow?
	Are radiologists biased by the AI’s predictions?
	What training is required for proper usage and bias avoidance?
Technical	How does the AI integrate into local IT infrastructure?
Economic	What is the direct cost of the AI (e.g., licensing)? Which other costs need to be considered (e.g., legal, training, etc.)
	Can return on investment be estimated and monitored?

## Section 7: What needs to be borne in mind to ensure long-term stability and safety of AI tools?

**Fig. 1:**
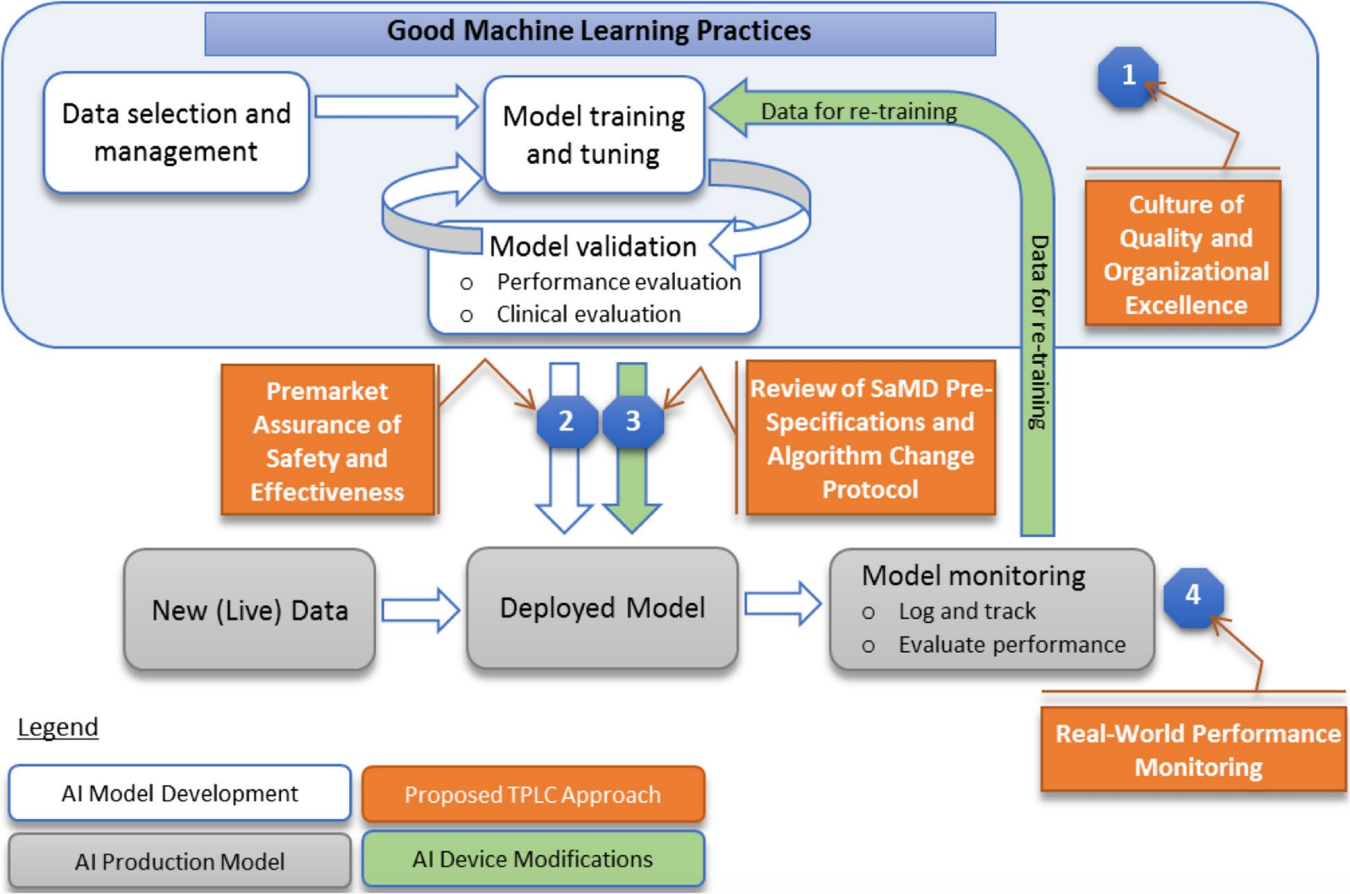
FDA’s planned Total Product Life Cycle (TPLC) approach to regulating AI/ML tools (from reference [63])

Monitoring the performance of AI models in clinical use is an important driver for safe and effective implementation of AI in clinical practice and is a key feature of the US Food and Drug Administration’s (FDA) Total Product Life Cycle approach (Fig. 1) to regulation of Software as a Medical Device (SaMD), which includes imaging AI [63]. End users should expect the performance of static (also known as “locked algorithms”) AI model performance to decline over time, due to shifts in local input data, changes to imaging equipment or protocols, acquisition software updates influencing source image parameters such as noise levels, or naturally occurring changes in patient populations and demographics [77]. Therefore, as the use of AI becomes more prevalent and the AI tools being deployed become more diverse, institutions using AI should establish ongoing performance oversight as one function of a local AI governance process [78]. Monitoring and a management strategy to ensure AI models are performing as expected over time are important as undetected performance degradation could have significant impact on patient safety and care [79–81]. In a potential future state where adaptive intelligence enables local model refinement, monitoring systems must be able to provide both baseline and longitudinal feedback information to continuously learning AI algorithms [77].

An ideal monitoring solution collects real time data on model performance, aggregates and analyses results comparing against expected performance at the local, regional or national benchmark level when feasible. However, this approach requires ready availability of ground truth and well defined performance benchmarks which is achievable today with some use cases and algorithms but not others. One common approach with triage type AI models that are tuned to identify findings which are also reported by radiologists would be an analysis of concordance or discordance between radiologist reports and model inference output. This approach may not work for quantitative outputs which cannot easily be reproduced by humans at scale or risk scores where validity can only be determined by analysis of longitudinal clinical data. Other targets for monitoring include changes in input metadata (e.g. equipment manufacturer, magnetic field strength or number of CT detectors), other relevant examination parameters and relevant demographic data about individual patients, since deviation of any or several of these from manufacturer specifications can result in degraded performance. It is incumbent on the local AI oversight group to determine on a case by case basis what sufficient monitoring looks like in a particular algorithm. In all cases, but especially when using quantitative models, radiologists may be able to determine the general validity of the AI output by confirming the absence of relevant imaging artifacts that would interfere with AI processing.

Strategies for real-world monitoring of AI in clinical practice should take into account the type of AI model being used and the risk to patient safety if the model performance declines. It will be important for the imaging community to establish monitoring approaches which can combine model output with appropriate forms of longitudinal analysis (with future imaging or EHR derived data or combination), through comparison to other clinical biomarkers of the same disease process, and with benchmark performance data from use of the same algorithm in a range of patients and a multitude of institutions. It should be noted that for almost all AI models with current regulatory approval, the model inference serves to augment, not replace, the radiologists’ interpretations, and therefore, patient-specific model failures of diagnostic or triage software are typically identified by the user before radiological reports are finalized and patient care initiated. However, when unsupervised autonomously-functioning AI algorithms emerge, robust monitoring solutions will be required to ensure patient safety. In future autonomous AI implementation, thorough understanding of failure modes and associated safety net processes may become paramount. This is further explored in Section 8. While we expect most developers of commercially-available AI solutions to be actively engaged in developing mechanisms for monitoring the effectiveness of their products, currently we are unaware of specific regulatory requirements of manufacturers for longitudinal AI performance monitoring, often referred to by regulators as post-market surveillance. As a result, nascent monitoring solutions are not standardized. Depending on the model and risk to patient safety a variety of monitoring strategies could be employed, ranging from real time continuous monitoring to periodic monitoring. Institutions should develop a clearly defined escalation and resolution strategy when monitoring detects model failure or performance drift occurs that defines the notification and action plan, and the mode of operation while the model performance is being assessed and the cause of model failure determined. In all cases, the monitoring strategy is predicated chiefly on the feasibility of well-defined performance parameters or a readily-available comparator (such as benchmark or ground truth).

### Periodic monitoring of model performance

Re-evaluation of AI model performance using updated local data sets at specified intervals, but at least annually, may be an appropriate monitoring mechanism for models where gathering real-time data on model performance is limited (quantification, workflow enhancement, etc.) or in instances where patient safety will not be immediately impacted [80]. Such a system requires that a new up-to-date evaluation data set be created using an appropriate number of clinical cases and parameters similar to the initial validation set to re-evaluate the performance of the model under current conditions. While this type of performance monitoring system could be useful for many of the AI models in clinical use, limitations include the time delay between ongoing use of the model and the occurrence of the discrete monitoring activity, which delays the institutions’ ability to take corrective actions should degradation occur. Another specific scenario which may require a re-evaluation of a model’s performance would be the issuance of a new model version by the manufacturer. It cannot be simply assumed by user sites that intended benefits introduced by a manufacturer’s deployment of the latest version of a model automatically generalize to the local practice.

### Monitoring for causes of data drift affecting model performance

Since changes in equipment, protocols and naturally occurring changes in population demographics are known causes of input data (source image) drift and potentially reduced AI model performance, institutions could elect to define baseline input data characteristics at the time of model acceptance and then monitor for data drift against that baseline state specific to each AI model [82]. Identification of relevant changes in input parameters could trigger the re-evaluation process described above for periodic monitoring. By monitoring for individual components of data drift, institutions could trigger re-evaluation of model performance depending on timing and severity of changes and initiate appropriate steps to safeguard patient care.

### Continuous monitoring of model performance

Strategies for continuously monitoring AI model performance cover many of the risks which should be evaluated before AI model introduction (outlined in Section 6 above). While real-time determinations of statistical parameters such as sensitivity, specificity, and positive predictive value are not possible during continuous monitoring, the algorithm’s performance compared to the interpreting radiologist’s final report, where possible, can be used as a surrogate for model accuracy. As explained above, harvesting of metadata about the examination should form part of this monitoring, and should include equipment manufacturer, protocol used, radiation dose and patient demographics. When available, the contemporaneous radiologist interpretation is considered a surrogate for ground truth, but the strength of this opportunistic labelling may be different from labelling provided during initial validation studies. Ideally this data collection could occur in the background, comparing information automatically extracted via suitable natural language processing methods against the radiologists’ reports as an AI accuracy measure, and data contained in the DICOM header to monitor the compliance of examination parameters with AI manufacturer specification of input data whenever feasible. Limited patient demographic information may also be found in the DICOM header and should be incorporated in the data collection. More robust monitoring and bias detection solutions may require expanded patient demographics. Continuous AI monitoring offers several advantages over episodic re-evaluations. Relevant information about AI model performance should be recorded in a dedicated AI data registry that allows generation of reports across multiple sites and geographies. Such benchmark data may be useful to individual sites as well as to the AI vendors [79]. At the local level, registry reports would allow institutions to identify performance degradation within their own local environment and could enable a systematic evaluation of the sources of potential data drift on a near real-time basis. For example, an institution with multiple CT scanners in their clinical workflow might identify performance degradation relative to their own historic performance in an AI model designed to detect intracranial hemorrhage. Hypothetically, analysis of the aggregate institutional registry data might show the poor performance to be limited to a single machine. Further analysis might also show that the performance degradation occurred after a software upgrade to that machine or change in examination protocol. Systematic analysis of cases that are not processed represent another important monitoring target. Such cases may point to systematic or anecdotal failure in the data acquisition or data transfer, impeding intended AI inference and preventing downstream clinical action to benefit from the same. Monitoring for non-performance represents an important building block of a local quality assurance system for clinical AI, which will be increasingly important as dependency of the clinical enterprise on AI increases in the future.

Aggregation of data from multiple institutions using the same AI models could provide information to developers to identify performance gaps that can be addressed in future versions of the algorithm, as well as meeting any future post-market surveillance regulatory requirements. While none of the AI models in clinical use employ continuous learning as a means for model improvement or local tuning, a hypothetical advantage of continuous monitoring solutions is the ability to inform future adaptive AI models with additional training data for continuous learning. However, there are significant limitations to the approach of continuous monitoring. Today such solutions may not be applicable for many (if any) AI models, including those performing quantitative tasks, and other AI models where performance cannot be measured real-time. Furthermore, continuous monitoring requires integration of production systems within a given institution, including information that may not be accessible to a manufacturer without local assistance and requisite infrastructure. Standards for this, specific regulatory guidance and the IT infrastructure for AI registries do not widely exist, and developing internal continuous monitoring solutions is likely to be cost- and resource-prohibitive for most institutions. Pilot projects for AI registries are underway; better understanding of the importance of aggregation and analysis of AI performance signal over time is likely to increase end-user interest in registry participation and may be a cost effective option to support this cause. However, in the absence of any regulatory requirements or availability of continuous learning AI models, demand may be limited. Currently, there are few AI models in limited markets that have regulatory approval for autonomously functioning AI [83], and the parameters for and frequency of evaluating model performance have yet to be determined. These parameters will vary with the disease process being evaluated, the risk to the patient in the event of model failure, and the prevalence of the disease in the target population. Therefore, one could imagine that performance monitoring could include intermittent random sampling of a pre-determined number of cases with ground truth comparison to spot-monitor performance over time.

### Future local tuning and continuous learning AI algorithms

Local tuning of AI models and continuous-learning AI algorithms prior to deployment have theoretical potential to improve the local performance of AI products. However, to date all AI tools which have received regulatory approval are static and cannot be locally tuned or undergo modifications using adaptive learning techniques. Recently, the US Food and Drug Administration (FDA) has released draft guidance for a “Predetermined Change Control Plan” [84] that would allow future modification to commercial algorithms for both local tuning and continuous learning. Any change control plan must include robust real-time AI model performance and measures that mitigate patient risk. Currently, this guidance has not been implemented but would be for models that are in the process of obtaining approval rather than those already approved.

### Other considerations: AI governance, managing technology lifecycle and local user environment

Given the complexity of managing all aspects of the AI lifecycle in clinical environments, provider entities engaging in the use of clinical AI are well served by formalizing local AI governance oversight and associated processes [78]. This is needed to deal with the many challenges in all phases of the AI product life cycle, which include procuring well-functioning AI, monitoring its performance over time, making adjustments to the local environment (e.g. scanner protocols, AI orchestration, device configuration, workflow integration including opportunistic capture of ground truth labels, etc.) over time as needed, and an orderly process to replace currently deployed products with future updates or alternative products. Often forgotten, but no less important, are the effects of the ever-more prevalent staff turnover amongst clinical end-users, radiologists and technical staff, including informaticists. As new users arrive in a local practice, they need to be properly assimilated, oriented, and trained in the available AI tools and associated work processes, to become effective participants in this technology-assisted care delivery paradigm. Ensuring that all local stakeholders are up to date and competent in the use of AI technology is a shared responsibility between vendors and the leaders of local institutional governance and oversight.

### Take-home points

Monitoring the performance of AI models in clinical practice is needed to ensure that any performance degradation is identified early so that appropriate measures can be taken to ensure patient safety. At a minimum, yearly re-evaluation of the need to assess model performance should be conducted, with monitoring of parameters known to be associated with drivers of input data drift. The need for more frequent re-evaluations should also be considered based on patient risk in the event of model failure and clinical decision relevance of a specific AI output. While not applicable to all AI models and clinical practices, continuous AI monitoring that captures model performance, examination parameters and patient demographics in data registries offers significant advantages over periodic re-evaluation of AI models, including real-time identification of local causes of diminished performance and providing developers with aggregated data for model improvement. Robust continuous performance monitoring will be needed prior to deployment of any autonomously functioning AI algorithms and is also a requisite for continuously-learning AI models.

#### Key statements—Long-term stability & safety of AI tools

- Naturally occurring data drift will cause AI model performance to degrade over time and should be anticipated by end-users.
- Monitoring strategies should include at minimum yearly re-valuation the performance of all AI models being used in clinical practice so that appropriate measures can be taken to ensure patient safety.
- Monitoring for changes in parameters known to be associated with input data drift could trigger more frequent re-evaluations.
- Continuous AI monitoring solutions that capture model performance, examination parameters and patient demographics in data registries that provide reports to end-users and developers offer significant advantages over periodic re-evaluation of AI models.
- Robust continuous performance monitoring will be needed prior to deployment of any autonomously functioning AI algorithms and required for continuous-learning AI models.

## Section 8: How can we assess whether (fully or partially) autonomous AI is likely/appropriate/safe in a particular clinical setting?

There are two distinct scenarios of AI implementation within radiology: augmentative AI and autonomous AI, each presenting unique considerations requiring rigorous scrutiny from both safety and ethical standpoints in the context of patient care.

### Augmentative AI

In this scenario, radiologists collaborate with AI systems to enhance diagnostic accuracy and drive efficiency. This collaboration provides an opportunity to increase the value provided by radiologists, but is not without challenges. As discussed in Section 6 (Human-AI Interaction), one crucial issue is the potential introduction of human–computer biases into the radiologic interpretation [59, 70–73]. These biases need to be both clarified and managed to ensure the AI’s output does not negatively influence the radiologist’s judgment. There are two general types of bias that can be introduced in a human–computer system, over-reliance, and under-reliance. Over-reliance, also known as automation bias increases the risk of False Positive (FP) and False Negative (FN) results: if the AI is right most of the time, radiologists may stop verifying the outputs, or come to trust the AI more than their own judgment. In this scenario the radiologist will accept incorrect AI results. Under-reliance has the same effect for the opposite reason. If the radiologist does not trust the AI results, they may disregard accurate AI output, also increasing FP and FN results. Ultimately, the output of the combination of radiologist plus the AI system must be optimized. These challenges can be further compounded by negative workplace attitudes [85] and factors that decrease personal perception of accountability [86] including radiologist burnout, and high workloads, both currently ubiquitous in radiology practice.

A robust approach to mitigating biases and challenges related to reliance involves continuous radiologist education about AI capabilities and limitations. Providing comprehensive information about AI decision-making, its results, and confidence levels can enhance transparency and help radiologists make informed judgments. In addition, categorizing scenarios where AI assistance may falter, and integrating that information into a robust training program, can empower radiologists to recognize and rectify errors. The accuracy of the AI system also affects rad-AI bias—bias is decreased by more accurate AI results [87]. Hence, identifying the most accurate AI model has clinical relevance. Finally, the measurement of rad-AI accuracy, along with directed feedback, can further refine the system's performance.

Ethical considerations surrounding augmentative AI are multifaceted. In settings where subspecialty radiologist coverage is limited, the introduction of AI assistance can significantly impact patient outcomes. However, the reliance on AI may lead to a dilemma where the presence of AI might influence the allocation of resources for training and retaining subspecialists. Careful consideration is needed to balance the ethical implications of AI augmentation in resource-constrained environments.

### Autonomous AI

In contrast to augmentative AI, autonomous AI operates without direct human oversight, making independent diagnostic decisions. This scenario raises heightened safety and ethical considerations [11]. Autonomous AI should be subject to stringent performance standards and comprehensive and continuous testing to ensure its reliability and accuracy. It is essential to critically assess the system's failure modes, considering that statistics from regulatory approval or vendor-provided accuracy rates might not adequately reflect real-world performance across various environments.

For autonomous AI, a rigorous ongoing monitoring program is imperative to detect and rectify errors promptly [11]. Training healthcare professionals in recognizing failure modes and offering a simple mechanism to disable autonomous AI when necessary is essential to avoid unchecked errors that could jeopardize patient care. Holistic continuous AI accuracy monitoring mechanisms are not yet mature. However, relying on such an a posteriori system to detect errors means that AI models may continue to provide inaccurate results for a period before there is sufficient data to confirm these inaccuracies. To gain earlier insights into AI's accuracy, additional tools for assessing expected AI outcomes based on input data (e.g., determining whether the input data falls within or outside the training data distribution) or comparing the results of one AI model to those of other AI models simultaneously can be employed [88].

Autonomous AI should be designed to initiate actions that are transparent, identifiable, and discoverable. The capacity to disable the AI system swiftly and effectively in the event of failure is crucial for patient safety. A streamlined process to address and mitigate failures should be in place to prevent repeating mistakes.

In communities where radiology services are scarce, the deployment of autonomous AI raises complex ethical questions. While autonomous AI can provide diagnostic insights in the absence of skilled radiologists, decisions made by AI systems could potentially lack nuanced human judgment. Striking a balance between accessible healthcare and maintaining diagnostic quality becomes a critical ethical concern.

Ultimately, the successful implementation of AI in radiology relies on an understanding of its implications, and proactive measures, including radiologist education, AI explainability, and radiologist-AI accuracy monitoring to address safety and ethical concerns.

## Section 9: Conclusion

Artificial intelligence in radiology is here to stay. It has the potential to add significant value to our care for patients, and to expand the horizons of what imaging can offer. Radiomics, for example, is an expanding field of data extraction and analysis that could not exist without AI.

As this exciting new technology increases its penetration and impact in healthcare, it is vital that it do so in a manner that is safe, and directed entirely towards benefit. Development, promotion and clinical adoption of AI tools must be aligned with benefit for those on whom these tools will be used [89]. Inevitably, commercial interests must be considered when developing and adopting AI tools, but these interests should not take primacy.

In this multisociety paper, we have endeavoured to provide guidance for developers, purchasers and users of AI in radiology to ensure that the practical issues that surround all stages of AI from conception to long-term integration in healthcare are clear, understood and addressed, and that patient and societal safety and well-being are the primary drivers of all decisions.

## Data Availability

Not applicable.
